# Comprehensive strategies for paclitaxel production: insights from plant cell culture, endophytic microorganisms, and synthetic biology

**DOI:** 10.1093/hr/uhae346

**Published:** 2024-12-12

**Authors:** Jia-Yuan Yin, Meng Lai, Xiao-Ying Yu, Ding-Ding Su, Xing-Yao Xiong, Yan-Lin Li

**Affiliations:** College of Horticulture, Engineering Research Center for Horticultural Crop Germplasm Creation and New Variety Breeding (Ministry of Education), Hunan Mid-Subtropical Quality Plant Breeding and Utilization Engineering Technology Research Center, No.1 Nongda Road, Furong District,Hunan Agricultural University, Changsha 410128, China; College of Horticulture, Engineering Research Center for Horticultural Crop Germplasm Creation and New Variety Breeding (Ministry of Education), Hunan Mid-Subtropical Quality Plant Breeding and Utilization Engineering Technology Research Center, No.1 Nongda Road, Furong District,Hunan Agricultural University, Changsha 410128, China; College of Horticulture, Engineering Research Center for Horticultural Crop Germplasm Creation and New Variety Breeding (Ministry of Education), Hunan Mid-Subtropical Quality Plant Breeding and Utilization Engineering Technology Research Center, No.1 Nongda Road, Furong District,Hunan Agricultural University, Changsha 410128, China; Institute of Advanced Agricultural Sciences, Peking University, No. 699 Binhu Road, Xiashan Ecological Economic Development Zone, Weifang 262041, China; College of Horticulture, Engineering Research Center for Horticultural Crop Germplasm Creation and New Variety Breeding (Ministry of Education), Hunan Mid-Subtropical Quality Plant Breeding and Utilization Engineering Technology Research Center, No.1 Nongda Road, Furong District,Hunan Agricultural University, Changsha 410128, China; Yuelushan Laboratory, The gathering area of Yuelushan Laboratory on Hongqi Road in Furong District, Changsha 410128, China; College of Horticulture, Engineering Research Center for Horticultural Crop Germplasm Creation and New Variety Breeding (Ministry of Education), Hunan Mid-Subtropical Quality Plant Breeding and Utilization Engineering Technology Research Center, No.1 Nongda Road, Furong District,Hunan Agricultural University, Changsha 410128, China; Institute of Advanced Agricultural Sciences, Peking University, No. 699 Binhu Road, Xiashan Ecological Economic Development Zone, Weifang 262041, China; Yuelushan Laboratory, The gathering area of Yuelushan Laboratory on Hongqi Road in Furong District, Changsha 410128, China

## Abstract

*Taxus* L*.*, an important ornamental, economic, and medicinal plant, is renowned for producing paclitaxel (Taxol®), a potent chemotherapeutic agent. The biosynthesis of paclitaxel involves intricate biosynthetic pathways, spanning multiple enzymatic steps. Despite advances, challenges remain in optimizing production methods. Microorganisms, particularly endophytic fungi, show potential in producing paclitaxel, though with limitations in yield and stability. The suspension culture of Taxus cells is a promising alternative, offering sustainable production, yet it requires further genetic and environmental optimization. Recent advancements in synthetic biology have enabled partial reconstitution of paclitaxel pathways in microbial and plant chassis. However, achieving complete biosynthesis remains an ongoing challenge. This review consolidates recent progress in paclitaxel biosynthesis, highlighting current limitations and future prospects for industrial-scale production.

## Introduction

*Taxus* L., a genus within the Taxaceae family, is renowned not only as an important ornamental tree species and a source of economic forest material, but also as a medicinal plant resource. Paclitaxel (trade name: Taxol) is a natural tetracyclic diterpenoid alkaloid isolated from the bark of Taxus in the 1960s and 1970s. In 1971, the structure of paclitaxel was first elucidated and subsequently applied in various cancer treatments due to its unique ability to stabilize and promote microtubule polymerization. Beyond oncology, paclitaxel’s therapeutic applications have expanded to include treatments for multiple sclerosis, intractable psoriasis, certain viral and fungal diseases, and rheumatoid arthritis, as well as other common diseases. With the introduction of various new formulations, such as oral liquids and topical gels, and their application in medical devices like heart stents, the global market for paclitaxel-based drugs has grown significantly. The constantly increasing new application pathways are expanding the market size of paclitaxel drugs from $4.51 billion in 2021 to a projected $11.16 billion by 2023 [1].

Initially, paclitaxel was extracted from the bark of *Taxus* species, a method that severely depleted wild Taxus populations. Consequently, environmental protection laws in many countries have prohibited this practice, prompting the search for alternative production methods. Although the chemical total synthesis of paclitaxel was achieved in 1994, the high production costs rendered it commercially unviable. As a result, production methods shifted to semisynthetic approaches, using branches and leaves to extract precursor substances, and semisynthetic methods gradually became the mainstream in the market [2]. However, semisynthetic strategies heavily rely on natural resources and are limited by slow growth of Taxus cells or leaves, which cannot meet the growing market demand. An alternative method for industrial-scale production involves the use of suspension cultures of Taxus cells for fermentation, and Python Biotech is the largest producer of paclitaxel using this method [1].

The biosynthetic pathway of paclitaxel has been elucidated, encompassing >10 enzymatic steps. The critical enzymes within the pathway have been thoroughly characterized, paving the way for biotechnological production. To date, heterologous reconstitution of paclitaxel precursor substances has been realized in species such as tobacco and *Escherichia coli*. Simultaneously with the discovery of paclitaxel in *Taxus chinensis* plants, numerous endophytic bacteria within the *Taxus* genus and species beyond were found to possess the capability to produce paclitaxel, enabling the heterologous synthesis of paclitaxel [1].

Given the widening gap between supply and demand for paclitaxel, biosynthetic production is emerging as a promising approach for its industrial-scale synthesis. Synthetic biology technology offers an ecofriendly and sustainable approach to the large-scale production of rare natural products by strategically constructing and optimizing the biosynthetic pathways of target compounds within chassis cells. This review aims to provide a comprehensive summary of the developmental history and recent advancements in paclitaxel biosynthesis and review the known biosynthetic pathways and transcriptional regulatory networks of paclitaxel, with a focus on the evolution of various synthesis methods and their respective advantages and limitations. By addressing the challenges in paclitaxel production, this review seeks to contribute to the theoretical foundation and future direction of its industrial synthesis.

### The biosynthetic pathway of paclitaxel

**Figure 1:**
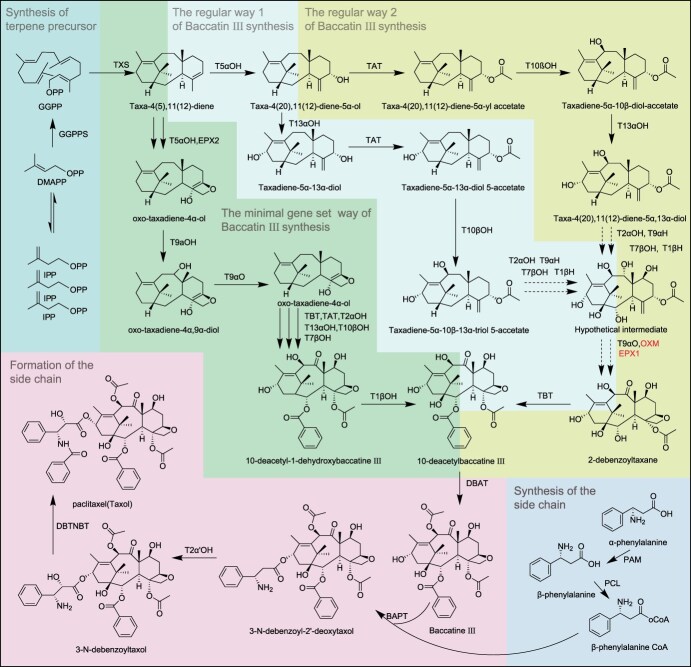
The proposed biosynthetic pathway of Taxol.

Currently, the intracellular biosynthesis of paclitaxel is understood to involve three primary pathways: two intracellular synthesis pathways in the Taxaceae family of plants and one heterologous reconstruction minimal gene set pathway in *Nicotiana benthamiana* (Fig. 1). The known biosynthetic pathways of paclitaxel contain three biosynthetic stages—the synthesis of terpene precursor, the synthesis of baccatin III, and the assembly of side chains and baccatin III. The above pathways involve 20 enzymes, including 18 characterized and 2 undiscovered enzymes (Table 1) [19, 20]. At present, the exploration of paclitaxel biosynthesis pathways and identification of key enzyme genes are mainly carried out in Taxaceae plants and *N. benthamiana*. The mechanism of paclitaxel synthesis in microorganisms is not yet clear, except for the paclitaxel synthesis strain constructed through microbial metabolic engineering.

**Table 1:** Key enzymes involved in the biosynthesis of paclitaxel.

Abbreviation	Enzyme	Reference	GenBank number	Probable localization
GGPPS	Geranylgeranyl diphosphate synthase	Hefner, Ketchum, & Croteau, 1998 [3]	AF081514	Chloroplast
TXS	Taxa-4(5),11(12)-diene synthase	Croteau & R., 1996 [4]	AY364469.1	Chloroplast
TAT	Taxadiene-5α-ol-O-acetyl transferase	K. Walker, Schoendorf, & Croteau, 2000 [5]	AF190130	Cytosol
TBT	Taxane-2α-O-benzoyl transferase	K. Walker & R. Croteau, 2000 [6]	AF297618	Cytosol
DBAT	10-deacetylbaccatin III-10-O acetyl transferase	Kevin Walker & Rodney Croteau, 2000 [7]	AF193765	Cytosol
T13αOH	Taxadiene-13α-hydroxylase	Jennewein et al., 2001 [8]	AY056019	Endoplasmic reticulum
T10βOH	Taxane-10β-hydroxylase	S. Jennewein, Wildung, Chau, Walker, & Croteau, 2004 [9]	AY563635	Endoplasmic reticulum
BAPT	Baccatin III-3-amino-3- phenylpropanoyl transferase	Kevin Walker, Fujisaki, Long, & Croteau, 2002 [10]	JF338879.1	Cytosol
DBTNBT	N-benzoyl transferase	K. Walker, Long, & Croteau, 2002 [11]	AF466397	Cytosol
T2αOH	Taxadiene-2α-hydroxylase	Chau & Croteau, 2004 [12]	AY518383	Endoplasmic reticulum
T7βOH	Taxane-7β-hydroxylase	Kaspera & Croteau, 2006 [13]	AY307951	Endoplasmic reticulum
T5αOH	Taxadiene-5α-hydroxylase	S. Jennewein, Rm., Rm., & Croteau, 2004 [14]	AY289209	Endoplasmic reticulum
PAM	Phenylalanine aminomutase	K. D. Walker, Klettke, Akiyama, & Croteau, 2004 [15]	AY582743	Cytosol
PCL	β-phenylalanoyl-CoA ligase	Karla Ramirez-Estrada et al., 2016 [16]	KM593667	Cytosol
T2’αOH	Taxane 2′α-hydroxylase	Sanchez-Muoz, Perez-Mata, Almagro, Cusido, & Moyano, 2020 [17]	KP178208.1	Cytosol
EPX1	4,20-epoxidase	McElroy & Jennewein, 2018 [18]		Cytosol
EPX2	2-oxoglutarate-dependent dioxygenase	Y. J. Zhang et al., 2023 [19]		Cytosol
OXM	4,5-oxomutase	McElroy & Jennewein, 2018 [18]		Cytosol
T9αOH	Taxane 9α-hydroxylase 1	Y. J. Zhang et al., 2023 [19]		Endoplasmic reticulum
T9αO	Taxane 9α-dioxygenase	Y. J. Zhang et al., 2023 [19]		Endoplasmic reticulum
T1βOH	Taxane 1β-hydroxylase	Y. J. Zhang et al., 2023 [19]		Endoplasmic reticulum
T9αH	Taxane 9α-hydroxylase 1	Jiang et al., 2024 [20]		Endoplasmic reticulum
TOT1	Taxane oxetanase 1	Jiang et al., 2024 [20]		Endoplasmic reticulum

**Figure 2:**
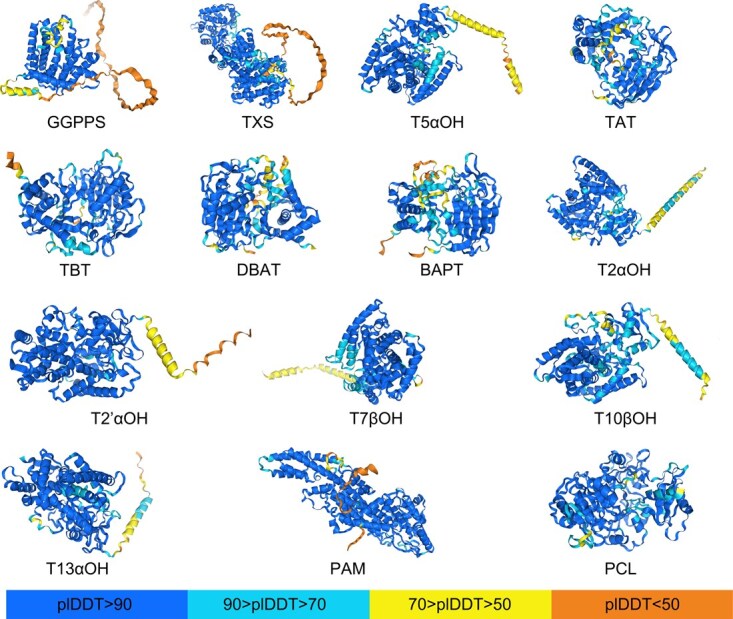
The protein structure prediction diagram was drawn by the author, and enzymes involved in the paclitaxel biosynthesis pathway with published sequences were selected for structure prediction using Alpha fold3

**Figure 3:**
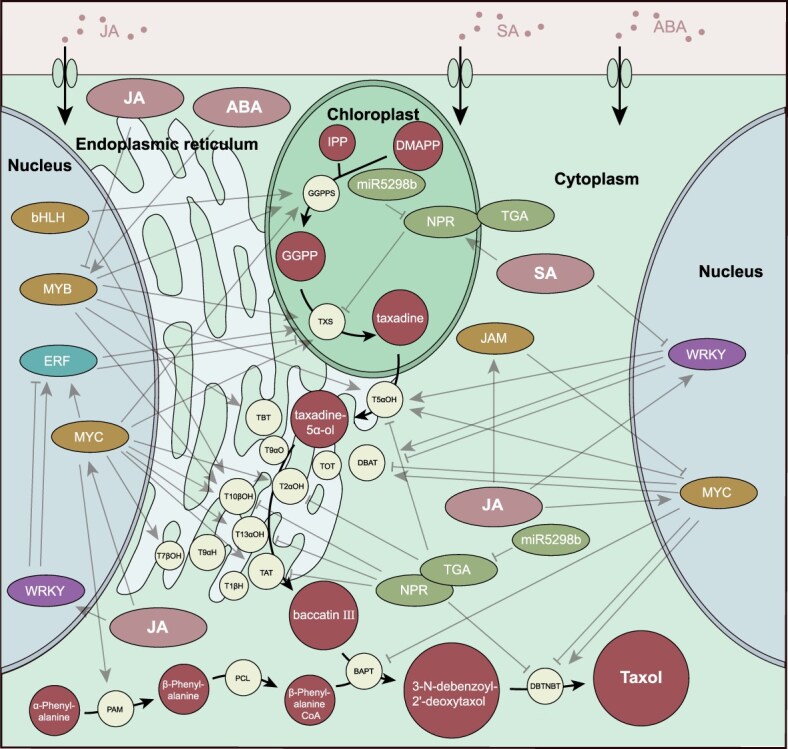
The proposed biosynthetic pathway of Taxol and known transcriptional regulatory networks

The first step in the paclitaxel biosynthesis pathway is the synthesis of terpene precursor，Here, geranylgeranyl diphosphate (GGPP) is synthesized through the catalytic action of geranylgeranyl diphosphate synthase (GGPPS), which facilitates the condensation of isopentenyl diphosphate (IPP) and dimethylallyl diphosphate (DMAPP). GGPP is then converted to taxadiene (taxa-4(5),11(12)-diene,15) in the chloroplasts by taxadiene synthase (TXS). From this point, the pathway diverges into two routes for the synthesis of baccatin III. In one pathway as described by Y. Zhang et al. (2023), taxadiene is transported to the endoplasmic reticulum (ER), where it undergoes hydroxylation at the C1, C2, C5, C7, C9, C10, and C13 positions, with further oxidation occurring at the C9 position. Through this sequence of modifications, which includes acetylation and the formation of the oxetane ring, baccatin III was synthesized. Another two-pathway shown by Jiang et al. (2024) shows that taxadiene is transported into the cytoplasm via a contact site between the plastid and ER. It undergoes synergistic catalysis by six membrane-bound oxidases (T2αH, T5αH, T7βH, T9αH, T13αH, and TOT) anchored within the ER, along with two cytoplasmic acyltransferases (TAT and TBT), ultimately leading to the formation of baccatine III [20](Fig. 2–3). After three pathways, the side chains formed by PAM and PCL in the cytoplasm are assembled with baccatine III through BAPT and further converted to paclitaxel through T2’α H and DBTNBT in the third stage.

In previous studies, genome research was typically conducted by combining multiple publicly available transcripts, which limited our understanding of the biosynthesis and regulatory mechanisms of paclitaxel. However, the recent publication of genomes for three *Taxus* species has overcome this limitation. The availability of these reference genomes has significantly improved the efficiency of identifying missing genes by providing precise gene localization, and it has facilitated the identification of two additional enzymes involved in paclitaxel biosynthesis—a bifunctional cytochrome P450 enzyme (taxane oxetanase 1, TOT1) and an enzyme responsible for the taxane oxidation of the C9 position (T9 alpha H1, T9αH)—thereby improving the known biosynthetic pathways, thereby refining the known biosynthetic pathways [20].

The biosynthetic pathway map of paclitaxel compiled and drawn by the author, with the minimal gene set way of baccatin III synthesis cited from Zhang’s research [19]. The regular way 1 and 2 of baccatin III synthesis were cited from Jiang’s research [20]. The enzymes in black font in the path have been successfully characterized, while the enzymes in red font have not been successfully characterized yet.

### Transcriptional regulatory network of paclitaxel biosynthesis

Plant hormones such as jasmonic acid (JA), gibberellins (GA), salicylic acid (SA), ethylene (ET), and abscisic acid (ABA) regulate the biosynthesis of paclitaxel to maintain a balance between the growth and defense response of *T. chinensis* [21]. During the intricate process of hormone regulation, transcription factor (TF) belonging to diverse families have emerged as crucial mediators, orchestrated by hormone signaling to facilitate paclitaxel biosynthesis. Currently, the regulatory pathways of GA and ET are still unclear. The signaling pathways that have been elucidated to play pivotal roles include JA, SA, and ABA (Fig. 3).

Within the intricate JA signaling cascade, TFs belonging to the MYC, JAM, WRKY, ERF, and MYB families orchestrate their responses to the initiated signal. Within the MYC family, the overexpression of *TcJAMYC1*, *TcJAMYC2*, and *TcJAMYC4* specifically downregulates a myriad of pathway genes in *T. chinesis* cells when exposed to JA (Table 2). Conversely, *TmMYC2*, *TmMYC3*, and *TmMYC4* exhibit transcriptional activation abilities, positively influencing the promoters of multiple pathway genes [27]. This regulation can also be indirect, such as *TcMYC2a* can directly or through ERF TFs regulate paclitaxel biosynthesis, while two AP2/ERF TFs, *TcERF12* and *TcERF15*, function as negative and positive factors, respectively, by binding to the GCC-box in the JA response element of the *TcTASY* promoter in *T. chinensis* [24, 33]. Interactions between MYCs and JAZs have been detected in *Taxus × media*，which shows MYCs can bind with JAZs to inhibit transcriptional activity [25]. MYB family member *TcMYB29a* downregulated the expression of T5αOH in response to JA signaling [21]. In the JAM family, JA signal-induced *TmJAM1* and *TmJAM2* significantly inhibited the selected paclitaxel synthesis pathway genes in this experiment, with *JAM2* showing a stronger inhibitory effect than *JAM1.* It is believed to be a feedback inhibition signal of the JA signaling pathway in *T. chinensis* [25]. In the WRKY family, *TcWRKY1* and *TcWRKY47* mainly play a role in upregulating pathway genes, while *TcWRKY47* also indirectly works by regulating TFs, upregulating *ERF15* while downregulating *ERF12* [30].

**Table 2:** List of TFs with known functions in the biosynthesis process of *T. chinensis*

Name	Signaling pathway	Positive regulation enzymes	Negative regulation enzymes	Positioning	Positioning method	Reference
*TmbHLH13*	Unknown	GGPPS T10βOH	Unknown	Nucleus	Subcellular localization	Yu et al., 2022 [22]
*TmbHLH46*	Unknown	GGPPS	Unknown	Nucleus	Prediction	Zhan et al., 2023 [23]
*TcERF12*	JA SA	Unknown	TXS	Nucleus	Prediction	M. Zhang et al., 2015 [24]
*TcERF15*	JA SA	TXS	Unknown	Nucleus	Prediction	M. Zhang et al., 2015 [24]
*TmJAM1*	JA	Unknown	DBTNBT T2αOH T7βOH T5αOH T13αOH DBAT T10βOH PAM GGPPS TXS BAPT	Cytoplasm	Prediction	Cui et al., 2019 [25]
*TmJAM2*	JA	Unknown	DBTNBT T2αOH T7βOH T5αOH T13αOH DBAT T10βOH PAM GGPPS TXS BAPT	Cytoplasm	Prediction	Cui et al., 2019 [25]
*TmMYC2*	JA	DBTNBT T2αOH T7βOH T5αOH T13αOH DBAT T10βOH PAM GGPPS TXS BAPT	Unknown	Cytoplasm	Prediction	Cui et al., 2019 [25]
*TmMYC3*	JA	DBTNBT T2αOH T7βOH T5αOH T13αOH DBAT T10βOH PAM GGPPS TXS BAPT	Unknown	Cytoplasm	Prediction	Cui et al., 2019 [25]
*TmMYC4*	JA	DBTNBT T2αOH T7βOH T5αOH T13αOH DBAT T10βOH PAM GGPPS TXS BAPT	Unknown	Cytoplasm	Prediction	Cui et al., 2019 [25]
*TcMYC2a*	JA	*TcERF12 TcERF15* DBTNBT TAT TXS T5αOH T13αOH	Unknown	Nucleus	Subcellular localization	M. Zhang, Jin, et al., 2018 [26]
*TcJAMYC1*	JA	Unknown	DBAT BAPT DBTNBT	Nucleus	Subcellular localization	Lenka et al., 2015 [27]
*TcJAMYC2*	JA	Unknown	T5αOH PAM DBAT BAPT DBTNBT	Nucleus	Subcellular localization	Lenka et al., 2015 [27]
*TcJAMYC4*	JA	Unknown	PAM DBAT BAPT DBTNBT	Nucleus	Subcellular localization	Lenka et al., 2015 [27]
*TmMYB3*	Unknown	TXS TBT	Unknown	Nucleus	Subcellular localization	Yu et al., 2020 [28]
*TmMYB39*	Unknown	GGPPS T10βOH	Unknown	Nucleus	Subcellular localization	Yu et al., 2022 [22]
*TmMYB17*	Unknown	TXS	Unknown	Nucleus	Prediction	Zhan et al., 2023 [23]
*TcMYB29a*	JA ABA	T5αOH	Unknown	Nucleus	Subcellular localization	Cao et al., 2022 [21]
*TcWRKY1*	JA	DBAT	Unknown	Nucleus	Prediction	S. Li, Zhang, Zhang, Fu, & Yu, 2013 [29]
*TcWRKY8*	SA	*TcERF15* DBAT T5αOH	TcERF12	Nucleus	Prediction	M. Zhang, Chen, et al., 2018 [30]
*TmWRKY12*	Unknown	Unknown	DBAT	Nucleus	Prediction	Zhan et al., 2023 [23]
*TcWRKY33*	SA	*TcERF15* DBAT	Unknown	Nucleus	Prediction	Chen et al., 2021 [31]
*TcWRKY47*	JA	*TcERF15* DBAT T5αOH	TcERF12	Nucleus	Prediction	M. Zhang, Chen, et al., 2018 [30]
*TcNPR3*	SA	Unknown	DBAT TXS T5αOH T2αOH T13αOH DBTNBT	Chloroplast	Prediction	Chen et al., 2023 [32]
*TcTGA6*	SA	Unknown	DBAT TXS T5αOH T2αOH T13αOH DBTNBT	Cytoplasm	Prediction	Chen et al., 2023 [32]

In the SA signaling pathway, TFs belonging to the ERF, WRKY, NPR, and TGA families respond to the signal (Table 2). *TcWRKY8* and *TcWRKY33* directly upregulated the expression of DBAT and T5αOH, while indirectly regulating paclitaxel synthesis by upregulating *ERF15* and downregulating *ERF12* [30]. *TcTGA6* physically interacts with *TcNPR3* to form a complex. *TcNPR3* upregulates in response to SA signaling, allowing *TcTGA6* to inhibit paclitaxel accumulation by directly binding to the TGACG motif downregulated pathway enzyme gene in the promoter. This inhibition can be relieved by *miR5298b* by cleaving *TcNPR3* [32]. In ABA signaling pathway, MYB family member *TcMYB29a* responded to the signal and upregulated the expression of T5αOH [21] (Table 2).

The regulatory network diagram was drawn by the author, and the path from GGPPS to baccatin III was cited from Jiang’s research [20]. The enzyme and TF positions in other parts were obtained from subcellular localization results and predicted results from http://www.csbio.sjtu.edu.cn/ (Table 1–2).

Some TFs with known regulatory targets have not been included in known signaling pathways. The bimolecular fluorescence complementation and yeast two-hybrid assays suggested potential interaction between *TmMYB39* and *TmbHLH13*, and transactivated the expression of GGPPS and T10βOH genes [22]. Through mass spectrometry imaging analysis, many novel cell-specific TFs involved in the biosynthesis of secondary metabolites have been identified, among which *TmbHLH46* upregulates GGPPS, *TmMYB17* upregulates TXS, and *TmWRKY12* downregulates DBAT [23]. Tissue-specific study across the stem of *T. media* identifies a phloem-specific *TmMYB3*, which participates in the biosynthesis of paclitaxel by activating the expression of TBT and TXS [28].

In addition, a large number of TFs have been found to be upregulated in hormone-induced transcriptome collections, mainly AP2/ERF, WRKY family, and bHLH family, but their targets are still to be identified. However, TFs are just one of the links in this vast regulatory network, and only a few TFs are considered to play an important role in regulating genes involved in the biosynthesis pathway of paclitaxel. There are still a large number of genes involved in hormone biosynthesis, and the regulatory mechanism of signal transduction is unknown.

### Microorganisms have been found to have the potential to produce paclitaxel

Partial endogenous microorganisms have been found to produce secondary metabolites similar to those of host plants, opening up new perspectives for the global drug market and pharmaceutical applications of biotechnology. *Taxomyces andreanae*, discovered in 1993, is an endophytic bacterium of *Taxus brevifolia* and is considered the first discovered fungus to produce paclitaxel. This discovery also marked the beginning of research on the production of paclitaxel by endophytic microorganism (Table 3). Since then, numerous studies have underscored the remarkable capacity of fungi belonging to diverse genera, including *Alternaria*, *Aspergillus*, *Cladosporium*, *Fusarium*, *Geotrichum*, and other genera (Table 3), in serving as viable producers of paclitaxel, showcasing their potential as significant contributors to this field.

**Table 3:** Discovered microorganisms producing paclitaxel and yield optimization methods

Genera	Species/Number	Host	Initial paclitaxel yield (μg/l)	Induction method	Final yield (μg/l)	Reference
*Alternaria*	*Alternaria alternata* MF5	*Taxus*	5700			Yang et al., 2018 [34]
	*Alternaria brassicicola* MVR1	*Terminalia arjuna*	140.8			Gill & Vasundhara, 2019 [35]
	*Alternaria tenuissima* TER995	*T. arjuna*	37.92	Bioprocess optimization	124.32	Gill & Vasundhara, 2019 [35]
	*A. alternata* F3	*Taxus cuspidata*	92.3	Add sodium acetate, SA, and silver nitrate	195.4	Fu et al., 2024 [36]
*Annulohypoxylon*	*Annulohypoxylon* sp.	*Taxus wallichiana* Zucc.	282.05			Ismaiel, Ahmed, Hassan, El-Sayed, & El-Din, 2017 [37]
	*Aspergillus flavipes* ATCC 24,487	Rhizosphere	185	Addition of fluconazole or *Podocarpus gracilior* leaf	320/210	Ashraf S. A. El-Sayed, Ali, Yassin, Zayed, & Ali, 2019 [38]
*Aspergillus*	*Aspergillus flavus* MW485934.1	Jojoba	88.6	Bioprocess optimization and γ-irradiation	375.9	Abdel-Fatah et al., 2022 [39]
	*A. fumigatus*	*Taxodium distichum*	84.41	Bioprocess optimization	307.03	Ismaiel et al., 2017 [37]
	TXD105					
	*A. fumigatus*	*Taxus* sp.	1590			Kumar, Singh, Thakur, Thakur, & Chand, 2019 [40]
	*Aspergillus oryzae*	*Tarenna asiatica*	95.04			A et al [41]
	*Aspergillus terreus* EFB108	*P. gracilior*	114.2	Physicochemical optimization and addition of surface-sterilized *P. gracilior* leaves	432	Ashraf S. A El-Sayed et al., 2018 [42]
	*Aspergillus aculeatinus* Tax-6	*T. chinensis var. maire*	334.92	Addition of CuSO4, SA, and sodium acetate	1337.56	Qiao, Ling, Yu, Huang, & Wang, 2017 [43]
	*A. aculeatinus* BT-2			Bioprocess optimization	560	Qiao, Tang, & Ling, 2020 [44]
*Cladosporium*	*C. cladosporioides* MD2	*T. media*	800			Peng Zhang, Zhou, & Yu, 2009 [45]
	*Cladosporium sphaerospermum* AUMC 6896	Clover leaf weevil	3.732	Adding ammonium acetate	30.365	Nagwa et al., 2018 [46]
*Epicoccum*	*Epicoccum nigrum* TXB502	*Taxus baccata*	61.35	Bioprocess optimization, γ-irradiation and immobilization	1364.63	E. S. R. El-Sayed, Zaki, Ahmed, & Ismaiel, 2020 [47]
	*Fusarium redolens*	*T. baccata*	70	Bioprocess optimization	198	Sanjog et al., 2014 [48]
	*E. nigrum*	*T. baccata*		Bioprocess optimization	57.1 ± 11.8	Somjaipeng, Medina, & Magan, 2016 [49]
*Fusarium.*	*Fusarium solani* Tax-3	*T. chinensis*	163.35			Deng, Liu, Chen, Ding, & Xie, 2009 [50]
	*F. solani* PQF9	*Podocarpus pilgeri*	18.2			Vu et al., 2023 [51]
*Grammothele*	*Grammothele lineata* SDL-CO-2015-1	*Corchorus olitorius*	382.2			Avizit et al., 2017 [52]
*Lasiodiplodia*	*Lasiodiplodia theobromae* SKJM1101	*P. nigrum*	247			Balendra, Kamalraj, & Jayabaskaran, 2017 [53]
*Metarhizium*	*Metarhizium anisopliae* H-27	*T. chinensis*	846.1			K. Liu, Ding, Deng, & Chen, 2009 [54]
*Metarizium*	*M. anisopliae* AUMC 5130	Clover leaf weevil	0.0023	Adding both ammonium acetate and salicylic acid	116.373	Nagwa et al., 2018 [46]
*Nodulisporium*	*Nodulisporium sylviform* HQD33	*T. cuspidata*	51.06–125.70	Strain improving	516.37	Zhao et al., 2008 [55]
*Paraconiothyrium*	*Paraconiothyrium variabile*	*T. baccata*		Adding salicylic acid	68.9 ± 11.9	Somjaipeng et al., 2016 [49]
*Penicillium*	*Penicillium chrysogenum* R16	*Glycin max*	170	Bioprocess optimization	250	A. El-Sayed, Enan, Al-Mohammadi, Moustafa, & El-Gazzar, 2020 [56]
	*Penicillium polonicum* AUMC14487	*Ginko biloba*	90.53	Bioprocess optimization and γ-irradiation	401.2	Abdel-Fatah, El-Batal, El-Sherbiny, Khalaf, & El-Sayed, 2021 [57]
*Pestalotiopsis*	*Pestalotiopsis hainanensis*	*Ailuropoda melanoleuca*	1466.87			Gu et al., 2015 [58]
	*Pestalotiopsis microspora*	*Taxodium mucronatum*	283.11	Addition of salicylic acid	625.47	Subban, Subramani, Srinivasan, Johnpaul, & Chelliah, 2020 [59]
*Phoma*	*Phoma medicaginis*	*T. wallichiana* var. *mairei*	1215			Zaiyou, Li, & Xiqiao, 2017 [60]
*Taxomyces*	*Taxomyces andreanae*	*T. brevifolia*	24–50			Stierle, Strobel, & Stierle, 1993 [61]

The sources of microorganisms producing paclitaxel are very diverse. In addition to endophytic microorganisms in Taxus plants, many other endophytic microorganisms isolated from plants have also been found to have the ability to produce paclitaxel, such as *Ginko biloba* and *Piper nigrum* (Table 3). Even animal pathogens have been found to have the ability to produce paclitaxel [58]. However, the biosynthesis pathway of paclitaxel in fungal cells is not yet clear. The weakened efficacy of paclitaxel production in endophytic bacteria is a common physiological phenomenon, According to reports, the initial yield of paclitaxel from *Cladosporium cladosporioides* MD2 isolated from *T. chinensis* was 800 μg/l, but after storage and passage of 5 years, it was only 5–7 μg/l, which means that endophytic bacteria may have a unique mechanism for synthesizing paclitaxel that is completely different from that of *T. chinensis* [43, 45].

Despite its wide range of sources, the concentration of paclitaxel in most microbial fermentation solutions is <1 mg/l, and the low yield of paclitaxel has always been a major challenge for microbial applications in industrial production [51]. To address this issue, various culture strategies have been adopted to increase the paclitaxel content produced by microorganisms, including three main pathways: (1) By adjusting the formula of the culture medium, the ratio of sugar source to carbon source, optimizing the aeration and stirring rate, and extending the fermentation period, the supply of nutrients and fermentation parameters can be optimized to promote microbial growth and metabolism. (2) Adding plant extracts or plant defense hormones to introduce chemical signals produced by host plants, as well as coculturing microorganisms and inducing their competition in living space and nutritional interactions, provide the necessary stimulation for microorganisms to produce paclitaxel. (3) Supplementing precursor substances, mineral ions, and trace elements, and adding metabolic inhibitors to block bypass pathways, promote the conversion of metabolic flux to paclitaxel, and regulate and maintain the balance of paclitaxel metabolism [62].

In addition to optimizing the cultivation conditions, gene manipulation significantly enhances the synthesis of paclitaxel by microorganisms. Nondirectional optimization includes mutagenesis breeding; the mutant K178 with high paclitaxel production was induced by UV radiation and diethyl sulfate (DES) in protoplasts, and its yield increased from 20 to 225.2 μg/l [63]. γ-irradiation results in a 2.42-fold enhancement of paclitaxel production in *Aspergillus fumigatus* TXD105 [64]. Targeted optimization can be achieved through gene recombination and knockdown or knockout of genes. Using genome recombination technology, Zhao et al. obtained three mutant strains with stable paclitaxel production and showed higher yields than the starting strain [55]. With the emergence of new synthetic biology technologies, such as CRISPR/Cas9-mediated genome editing, optimizing the relative orientation of bacterial strains can be achieved through precise manipulation of individual genes. On the sterol synthesis pathway，knockdown of the rate-limiting enzyme gene by using RNAi technology or knockout of the rate-limiting enzyme gene by using CRISPR/Cas9 technology can facilitate the metabolic flux to paclitaxel [65]. By optimizing the transcription levels of key genes via the alteration of promoter strength, codon usage and gene copy number, the accumulation of paclitaxel in endophytic bacteria due to the ectopic expression of GGPPS in *T. chinensis* can be enhanced [66]. In summary, discovering and transforming new strains for producing paclitaxel and optimizing the fermentation process remain the main goals in the field of paclitaxel microbial production.

### Production of paclitaxel through plant cell culture

At present, there are mainly two types of paclitaxel raw materials on the market: paclitaxel extracted from the bark of Taxus plants and Taxus cell culture medium, and semisynthetic paclitaxel ‘docetaxel’. The product Paclitaxel (Taxol®) Docetaxel semisynthetic (*Taxotere*) from Phyton Biotech Inc. (Germany) has obtained FDA approval, while Samyang Biopharm (Republic of Korea) produces Paclitaxel encapsulated with polymeric micelle technology through suspension culture of *T. chinensis* cells (Genexol®PM), which has also entered Phase II, indicating that suspension culture has become a successful industrial and commercial method for paclitaxel biosynthesis [67]. Compared to other biosynthetic methods, suspension culture has many advantages. Firstly, the entire production process can be completed in a bioreactor, and building a large-scale production platform allows for more production to be carried out simultaneously, which is environmentally friendly. Secondly, because the cells of *T. chinensis* have a complete pathway for paclitaxel metabolism and can proliferate in the reactor, the entire platform is ecological and sustainable. Finally, the high-yield cell lines generated by genetic manipulation combined with the use of multiple inducers can significantly increase yield, and these advantages also make it a new direction for the industrial production of paclitaxel after the semisynthetic method of extracting branches and leaves from *Taxus* plants. The concept of suspension culture of *T. chinensis* cells was first proposed in a patent application, with an initial yield of 1–3 mg/l [18]. After years of development, the current highest yield has been increased to 565 mg/l (Table 4). These strategies for increasing yield mainly include changing the culture medium and carbon source formula, optimizing culture conditions, using inducers to increase secondary metabolite synthesis, and gene-level operations.

**Table 4:** Yield-increasing strategy of suspension culture of *T. chinensis* cells.

Type	Method	Species	Initial production of Taxol	Regular production of Taxol	Reference
Medium and carbon source change	Two-phase culture and sucrose feeding	*T. chinensis*	5.2 mg/l	36.0 ± 3.5 mg/l	Wang et al., 2001 [68]
Medium and carbon source change	Repeated induction and addition of methyl jasmonate	*T. chinensis*		565 ± 47 mg/l	Zhen-Yu, WangJian-Jiang, & Zhong, 2002 [69]
Add inducer	Methyl jasmonate-induced	*T. media*		104.2 mg/l	Yukimune, Tabata, Higashi, & Hara, 1996 [70]
Add inducer	Coronatine (CORO)-induced	*T. media*	8.14 mg/l	77.46 mg/l	Onrubia et al., 2013 [71]
Add inducer	Cyclodextrin (CD)- and CORO-induced	*T. media*	5 mg/l (Total taxane)	35 mg/l (Total taxane)	K. Ramirez-Estrada et al., 2015 [72]
Add inducer	Add inducers prepared from endophytic bacteria	*Taxus yunnanensis*	2.65 μg/g DW	8.72 μg/g DW	Q. X. Liu et al., 2023 [73]
Add inducer	Add gold nanoparticles (AuNPs)	*T. baccata*		90.94 ± 13.20 μg/l	Golinejad, Mirjalili, Rezadoost, & Ghorbanpour, 2023 [74]
Add inducer	Add Ag+	*T. yunnanensis*	1.4 mg/l	39.8 mg/l	C. H. Zhang, Wu, & He, 2002 [75]
Add inducer	Add chitosan	*T. yunnanensis*	1.4 mg/l	39.8 mg/l	C. H. Zhang et al., 2002 [75]
Add inducer	Add salicylic acid, fungal elicitor and methyl jasmonate	*T. baccata*	2.45 mg/l	39.5 mg/l	A Yari Khosroushahi, Valizadeh, Ghasempour, Khosrowshahli, & Omidi, 2013 [76]
Add inducer	Add methyl jasmonate, salicylic acid, and inducers prepared from endophytic bacteria	*T. baccata*	2.45 mg/l	39.5 mg/g DW	A. Yari Khosroushahi et al., 2006 [76]
Gene manipulation	DBTNBT overexpression	*T. chinensis*	71.01 mg/l	310 mg/l	Perez-Matas, Hidalgo-Martinez, Moyano, Palazon, & Bonfill, 2024 [77]
Gene manipulation	Polyploid mutagenesis	*T. baccata*	2.5 mg/l (Total taxane)	21.64 mg/l (Total taxane)	Escrich et al., 2023 [78]
Gene manipulation	Overexpress the dbat gene coding for 10-deacetylbaccatin III-10 beta-O-acetyltransferase	*T. chinensis*	20 ± 0.6 μg/g DW	35 ± 0.6 μg/g DW	P. Zhang et al., 2011 [79]
Gene manipulation	BAPT overexpression	*T. chinensis*	71.01 mg/l	135 mg/l	Perez-Matas et al., 2024 [77]

Different carbon sources affect the growth of suspension-cultured cells and the synthesis of their own metabolites. Sucrose and fructose are beneficial for increasing paclitaxel production; the development of two-phase (aqueous–organic) cultivation techniques for *T. chinensis* has been reported to significantly enhance taxol yields by a factor of six, which was achieved when the two-phase cultivation system was supplemented with sucrose feeding, marking a notable advancement in the production of this valuable compound [68]. In addition to carbon sources, environmental factors such as temperature and gas composition in the culture medium can also affect the content and production rate of paclitaxel. High levels of carbon dioxide prevent the biosynthesis of paclitaxel, while promotion of paclitaxel synthesis can be observed at low oxygen levels, indicating that gas composition may affect the production of secondary metabolites and inhibit P450 enzyme activity by altering the distribution of nutrients [80]. During suspension culture, the sensitivity of cells to shear forces may lead to slow growth. This negative effect can be avoided by using agar, gel, polyacrylamide, hollow fiber membrane, polyurethane foam, and other matrices to encapsulate cells. *Taxus* cells fixed in calcium alginate were cultured in a stirred bioreactor and reached a peak yield of 43.43 mg/l taxol [81]. To optimize the cultivation conditions and meet the standards of industrial production, a large-scale bioreactor with a volume of 75 000 l has also been developed to increase the yield of paclitaxel [18]. Due to its own cytotoxicity, the accumulation of taxanes in the culture medium and cells can inhibit cell growth and metabolism, leading to production stagnation. After *in situ* removal of paclitaxel, baccatin III, and other taxane substances in the culture medium, an increase in paclitaxel production was observed [82].

Inducers enhance plant defense responses by activating multiple defense-related hormone signaling pathways, thereby increasing the content of secondary metabolites. This pathway can also be found in *T. chinensis* cells and validated through transcriptome sequencing. In the absence of reference genome data, differential transcription of paclitaxel biosynthesis pathway enzymes can be detected in cells induced by inducers. The multiple transcripts obtained using this method also facilitated the discovery of key enzyme genes and TFs in the biosynthesis pathway of paclitaxel. The currently discovered inducers mainly include JA, cyclodextrin, fungal extract, and nano metal particles (Table 4).

While optimizing the cultivation conditions and using inducing substances, genetic manipulation can increase the background paclitaxel content of cell lines and accelerate the efficiency of biosynthesis. The methods for further improving the biosynthesis yield of paclitaxel at the genetic level include polyploid mutagenesis, reducing the expression of rate-limiting enzymes, and enhancing the expression of key enzymes. The enzymes that were upregulated include pathway enzymes in the biosynthesis step of paclitaxel, which effectively increased the final paclitaxel production by enhancing the main synthesis branch (Table 4) and the redirection of flux away from competitive branches by antisense silencing of the T14βOH gene in *T. media* [83]*.*

### Genetic manipulation can increase the yield of paclitaxel biosynthesis

Due to the advantages of low sugar source cost, high extraction efficiency, and environmental friendliness of heterologous biosynthetic systems, utilizing microbial metabolic engineering and synthetic biology techniques to construct suitable heterologous biosynthetic systems for the production of paclitaxel has gradually become a research hotspot. Taxadiene and taxadien-5α-ol are a class of diterpenoid compounds and are also considered precursor substances for the biosynthesis of paclitaxel. Taxadiene is a diterpenoid compound derived from cyclized geranyl pyrophosphate (GGPP) substrate of taxadiene synthase (TS). GGPP is a common precursor produced from MEP or MVA pathway and generated by geranyl diphosphate synthase (GGPPS). Taxadiene is subsequently catalyzed and modified by taxadien-5α-hydroxylase (T5αOH) to produce various mono-oxygen and dioxygeal taxanes, as well as taxadien-5α-ol. The biosynthesis and modification of the two precursor substances mentioned above have been achieved in *E. coli* and *Saccharomyces cerevisiae* systems (Table 5). Subsequently, at least 17 enzyme-catalyzed steps are required to obtain paclitaxel, with some cytochrome P450s still awaiting overexpression and characterization [99].

**Table 5:** Partial genetic engineering construction of paclitaxel heterologous synthesis system

Host	Taxadiene yield	Control methods	Reference
*E. coli*	300 mg/l	IPTG induction	P. et al., 2010 [84]
*E. coli*	570 ± 45 mg/l	optimizing P450 expression, reductase partner interactions, and N-terminal modifications	Biggs et al., 2016 [85]
*E. coli*	1020 ± 0.08 mg/l	IPTG induction	P. et al., 2010 [84]
*E. coli*	7.0 mg/l	Optimized the linker for fusing taxadiene-5α-hydroxylase with its reductase partner cytochrome P450 reductase,	Wu, Huang, Wang, Yu, & Xu, 2022 [86]
Cocultivation of *E. coli* and *S. cerevisiae*	33 mg/l	Stable cocultivation in bioreactors	Zhou, Qiao, Edgar, & Stephanopoulos, 2015 [87]
*S. cerevisiae*	20 mg/l	YP-galactose medium culture	Amanda et al., 2017 [88]
*S. cerevisiae*	129 ± 15 mg/l	Multicopy chromosomal integration of TASY, shake flask	B. Nowrouzi et al., 2020 [89]
*S. cerevisiae*	184.2 mg/l	Initial glucose 20 g/l, supplemental glucose <1 g/l	C. L. Zhang et al., 2023 [90]
*S. cerevisiae*	22 mg/l	500 ml bioreactor, 250 rpm	Walls, Martinez, & Rios-Solis, 2022 [91]
*S. cerevisiae*	98.9 mg/l	Micro bioreactor and pH control	Walls et al., 2021 [92]
*S. cerevisiae*	33.3 ± 3.9 mg/l	500 ml of SDG medium containing 20 g/l galactose (separately autoclaved), 6.7 g/l yeast nitrogen base with ammonium sulfate and without amino acids and 0.79 g/l complete synthetic mixture, 600 rpm, lasted for 50 h (five residence times) for each steady state	Behnaz Nowrouzi, Torres-Montero, Kerkhoven, Martinez, & Rios-Solis, 2024 [93]
*Arabidopsis thaliana*	600 ng/g DW	Spraying with DEX	Besumbes et al., 2004 [94]
*Artemisia annua*	129.7 μg/g DW	TXS inserted into pCAMBIA1304, transformed into *A. annua*	M. Y. Li, Jiang, Yu, & Miao, 2015 [95]
*Panax ginseng*	9.1 μg/g DW	Transfer of TS gene	Cha et al., 2012 [96]
*N. benthamiana*	154.84 ng/g FW (baccatin III)	Kept in the light to dry the leaves (1 h), and then subsequently kept in the dark for 3–4 days at room temperature	Y. J. Zhang et al., 2023 [19]
*N. benthamiana*	56.6 μg/g FW	Chloroplastic compartmentalized metabolic engineering strategy, combined with enhancement of isoprenoid precursors	J. Li et al., 2019 [97]
*Lycopersicon esculentum*	160 mg/kg DW	Transgenic method redirects carotenoid metabolism	Kovacs et al., 2007 [98]

In *E. coli*, in 2010, Ajikumar et al. optimized the synthesis and metabolism balance of paclitaxel by designing a multimodule metabolic pathway, and achieved a paclitaxel production of 300 mg/l in shake flask fermentation. Then, by changing the feeding batch fermentation, the yield of paclitaxel was further increased to 1020 mg/l [84]. But when the author expressed the subsequent enzyme taxine-5 α - hydroxylase (a membrane-bound cytochrome P450), a 10-fold decrease in total taxane titers was observed. It is estimated that membrane-bound cytochrome P450 (such as taxine-5α-hydroxylase) accounts for about half of the 19 enzymatic steps in the biosynthesis pathway of paclitaxel, but the overexpression of P450 enzymes in *E. coli* is greatly hindered, making it difficult to complete subsequent steps [99]. The expression of T5αOH and its reductase partner in *E. coli* was optimized through N-terminal modification, achieving a 5-fold increase in the titer of oxygenated taxane, reaching 570 mg/l. This indicates that the prokaryotic system is also suitable for P450-based oxidative chemistry but requires careful regulation [85]. Due to the lack of a complete inner membrane system and protein post-translational modification system in the prokaryotic system, the synthesis of paclitaxel using the prokaryotic system is severely limited. Therefore, in recent years, there has been no significant breakthrough in the heterogeneous synthesis of paclitaxel in *E. coli* (Table 5).

Compared to prokaryotes, eukaryotic systems are considered potential candidates for the heterogeneous synthesis of paclitaxel, as eukaryotic systems are suitable for the expression of various heterologous P450 hydroxylases and their reductases. The eukaryotic host *S. cerevisiae* possesses the biosynthetic mechanisms required for the expression of P450 enzymes, including translocation through the ER and natural electron transfer mechanisms, which have been favored by researchers. At present, the content of paclitaxel obtained based on the *S. cerevisiae* system has reached to 184.2 mg/l (Table 5).

Due to the advantage of eukaryotic systems being suitable for the expression of various heterologous P450 hydroxylases and their reductases, partial transfer of the paclitaxel metabolic pathway into plant cells has been achieved, and the number of steps completed in plant cells continues to increase. Li et al. introduced taxadiene synthase and taxadiene-5α-hydroxylase in *N. benthamiana* through chloroplastic compartmentalized metabolic engineering to produce taxadiene (56.6 μg/g FW) and taxadiene-5α-ol (1.3 μg/g FW) [97]. By means of heterologous expression in *N. benthamiana*, Zhang et al. made use of both the oxomutases/epoxidases that had been characterized previously and the newly identified ones, taxane 1β - hydroxylase, taxane 9α - hydroxylase, taxane 9α - dioxygenase and phenylalanine - CoA ligase to successfully biosynthesize the key intermediate baccatin III, establish a metabolic route for taxoid biosynthesis, and determine the minimum set of genes required for paclitaxel biosynthesis in a plant host, thus providing broad prospects for the industrial synthesis of paclitaxel with plant cells as the substrate [19].

### Current methodological challenges and future directions

#### Microbial synthesis—different synthetic pathways and production stability challenges

Microbial systems, especially engineered endophytic microorganisms, exhibit considerable potential for paclitaxel production due to their adaptability and rapid growth rate. However, these microorganisms often face significant losses in paclitaxel productivity during storage and passage cultivation. The downregulation of paclitaxel gene expression in endophytic fungi following passage culture has been well documented, leading to significant challenges related to low yield and unstable production. These issues primarily arise from the incomplete understanding of the synthetic pathway of paclitaxel in microbial cells, which differs substantially from that in plant cells. Additionally, the low activity of key enzymes and inefficiencies in the synthesis of intermediate metabolites further contribute to the reduction in production. Although synthetic biology has enabled the development of recombinant strains capable of generating paclitaxel, the yields remain below the levels necessary for commercial viability.

To address these challenges, future research should focus on analyzing the distinct pathways of paclitaxel synthesis in microbial cells, identifying mechanisms underlying the reduction in production after multiple generations of cultivation, and enhancing the expression efficiency of key enzymes through genetic engineering. Discovering new strains that can independently produce paclitaxel, as well as maintaining the production capability of endogenous strains after being isolated from host plants, remains a primary research focus. Additionally, employing metabolic flux analysis can help optimize the overall biosynthetic pathway, improving both yield and production stability. The design of more efficient production platforms through synthetic metabolic engineering offers a promising approach to increasing output.

#### Plant cell culture—analyzing complex networks for precise control

Suspension culture of *T. chinensis* cells, due to their ability to independently produce paclitaxel and sustain production, is considered a key avenue for industrial synthesis of paclitaxel. However, scaling up production in bioreactors presents challenges related to maintaining optimal oxygen supply, nutrient composition, and precise control over metabolic processes. Variability in cell growth state and response to external inducers further complicates production, making it difficult to maintain consistency at an industrial scale. Moreover, early studies lacked high-quality reference genomes, resulting in only a partial understanding of the extensive metabolic regulatory network involved in paclitaxel biosynthesis. Many enzymes in the pathway and key TFs remain unidentified, which limits precise regulation of the synthesis process and hampers efforts to counter the decline in paclitaxel production due to epigenetic effects.

Exploring uncharted areas of the paclitaxel synthesis metabolic network, such as the roles of GA and ET hormone signaling pathways, will be essential for achieving precise regulation in industrial production. Future research should consider not only individual hormone signaling pathways but also the interplay among multiple hormone signals to guide products toward the main pathway of paclitaxel synthesis using strategic combinations of different inducers. Additionally, optimizing bioreactor design, along with precise control strategies like continuous and fed-batch cultivation, exploring the combined use of multiple inducers, refining culture conditions, and engineering cellular environments, such as developing microcarrier systems or coculture techniques, could significantly enhance cell metabolism and yield, and further improve production scalability.

#### Synthetic biology—maximizing production efficiency in prokaryotic and eukaryotic systems

Synthetic biology holds great promise for the industrial-scale production of paclitaxel by reconstructing biosynthetic pathways in heterologous hosts. However, this approach faces challenges such as pathway length, enzyme activity, and expression stability. Achieving high efficiency in these processes, particularly in microbial systems, remains a significant bottleneck. For instance, the lack of a complete inner membrane system and protein post-translational modification in prokaryotic systems impedes the effective characterization of multiple P450 enzymes involved in paclitaxel biosynthesis, slowing progress in heterologous synthesis using *E. coli* as a chassis. Conversely, eukaryotic systems like *S. cerevisiae* are more suitable for the expression of P450 hydroxylases and their reductases, making them advantageous for the synthesis of paclitaxel precursors. However, the precursors synthesized in *S. cerevisiae* still require multiple steps to convert into paclitaxel.

For microbial chassis cells, the advantage of producing paclitaxel lies in their rapid reproduction and high yield, rather than involving complex synthetic steps. Segmenting the paclitaxel synthesis pathway and reconstructing it in different microbial chassis cells to produce intermediates at various stages may leverage the efficiency of microbial production systems. On the other hand, plant chassis cells are capable of more extensive biosynthetic processes, and with the release of a minimal gene set for paclitaxel biosynthesis in plants, they may serve as future candidates for complete synthesis pathways. Additionally, genome editing technologies like CRISPR/Cas9 can be employed to streamline and enhance metabolic pathways, improving production efficiency. Integrating synthetic biology with systems biology and machine learning can further refine pathway design and control, facilitating easier scaling of production processes.

## Conclusion

The advancement of high-quality reference genomes has significantly enhanced the understanding of paclitaxel biosynthesis, aiding in the identification of key enzymes and regulatory networks. However, critical challenges persist in optimizing production, particularly due to low yield stability, complex metabolic pathways, and technical barriers in large-scale synthesis. Addressing these bottlenecks requires a multifaceted approach that combines genetic engineering, pathway optimization, and advanced bioprocessing techniques.

Future research should prioritize enhancing enzyme activity through targeted genetic modification, exploring the synergistic effects of multiple inducers, and developing more robust heterologous systems for scalable and efficient production. Furthermore, improvements in bioreactor design and culture conditions can significantly boost industrial feasibility. Continued innovation in these areas is essential to overcoming current limitations, enabling the sustainable and cost-effective production of paclitaxel at an industrial scale.

By focusing on these strategic areas, future efforts can make significant progress toward meeting global demand, ultimately driving both scientific discovery and practical applications forward.

## Data Availability

Data sharing is not applicable to this article as no new data were created or analyzed in this study.
